# Pregnancy in Patients With McArdle’s Disease

**DOI:** 10.7759/cureus.84658

**Published:** 2025-05-23

**Authors:** Joana Rodrigues dos Santos, Catarina Távora, Inês Nogueira da Fonseca, Diogo Cruz

**Affiliations:** 1 Internal Medicine, Hospital De Cascais, Lisbon, PRT

**Keywords:** inherited disorder of metabolism, mcardle’s disease, pregnancy, rhabdomyolysis, type v glycogenosis

## Abstract

The influence of hereditary disorders of metabolism in pregnancy can be unclear, probably due to their rarity. McArdle’s disease does not have an impact on fertility; therefore, some cases of pregnancy have been reported in patients with McArdle’s disease.

This article reviews published cases of pregnant women with McArdle's disease, focusing on the period of pregnancy, delivery, and postpartum. Following the Preferred Reporting Items for Systematic Reviews and Meta-Analyses (PRISMA), the authors conducted a literature search of PubMed, Web of Science, and Dialnet, as well as the journal Ata Portuguesa de Ginecologia e Obstetrícia (Journal of the Portuguese Societies of Gynecology and Obstetrics). The authors searched for articles in Portuguese, English, French, and Spanish using the combination of keywords “pregnancy” and any of the following: “McArdle's Disease”, “Type V Glycogenosis”, or “Myophosphorylase”, available as of February 18, 2023. Thirteen articles corresponded to the search criteria, describing a total of 26 patients with McArdle’s disease and 37 pregnancies (one of them a twin pregnancy). No case reports or case series were excluded.

The objective was to evaluate obstetric index, comorbidities, gestational age, description of pregnancy evolution, mode of delivery, intrapartum pharmacological measures, and clinical evolution in the postpartum period. Of the 16 cases in which age of the patient was mentioned, the mean age was 27.75 years. Of the 18 cases in which the obstetric index was mentioned, 13 (72.2%) pregnancies were first pregnancies and 5 (27.8%) were second pregnancies. In addition to McArdle's disease, some patients had other comorbidities: one had Crohn's disease; one had dilated cardiomyopathy; one had grade II obesity; one had arterial hypertension, dyslipidemia, gastroesophageal reflux disease, and grade III obesity; and one had recurrent tonsillitis and allergic rhinitis. The mean age of pregnant women was 27.75 years. In most cases (75.7%), there was no mention of complications during pregnancy. There were four (10.8%) cases of rhabdomyolysis (two triggered by infectious diseases, one by exercise, and one with no evident trigger). Two women developed gestational diabetes, one had pre-eclampsia, one patient with dilated cardiomyopathy had worsening complaints of heart failure, and one patient developed idiopathic thrombocytopenia, which resolved after delivery. Of the 18 patients questioned about tolerance to exercise, two (11.1%) reported worsening of myalgia during pregnancy, while the rest reported symptomatic improvement. In 16 cases, it was necessary to perform a cesarean section. The remaining 22 deliveries were vaginal. Of the 14 cases in which anesthesia was specified, 12 (85.7%) patients were given epidural and 2 patients were given general anesthesia. There were three reported cases of rhabdomyolysis after childbirth.

Pregnancy in patients with McArdle's disease does not seem to be associated with more complications than pregnancy in women without the disease, and there may even be an improvement of the symptoms during pregnancy.

This review will help physicians provide such patients with better counseling and take measures to prevent complications. Implementing protocols designed specifically for pregnancy, delivery, and postpartum in patients with McArdle's disease could be beneficial, reducing the episodes of rhabdomyolysis.

## Introduction and background

McArdle's disease (type V glycogenosis or myophosphorylase deficiency) is a hereditary disorder of metabolism, characterized by the absence of glycogenolysis ability in the skeletal muscle, leading to intolerance to physical exercise, with episodes of significant rhabdomyolysis [1]. The prevalence of McArdle's disease ranges from 1 in 50,000 to 1 in 200,000 in the United States [2]. Fertility does not seem to be affected by the disease; therefore, there are some reported cases of pregnant women with McArdle's disease.

Maternal metabolism changes substantially during pregnancy and the delivery can be seen as a vigorous, intense exercise.

With this article, the authors intend to review the published cases of pregnancy in patients with McArdle’s disease, with greater emphasis on the periods of pregnancy, delivery, and postpartum. The goal of this work is to understand pregnancy outcomes in patients with McArdle's disease. This will enable better counseling of patients of childbearing age, in order to plan a possible pregnancy, as well as allow better monitoring of pregnancy, childbirth, and the postpartum period.

## Review

Methods

**Figure 1:**
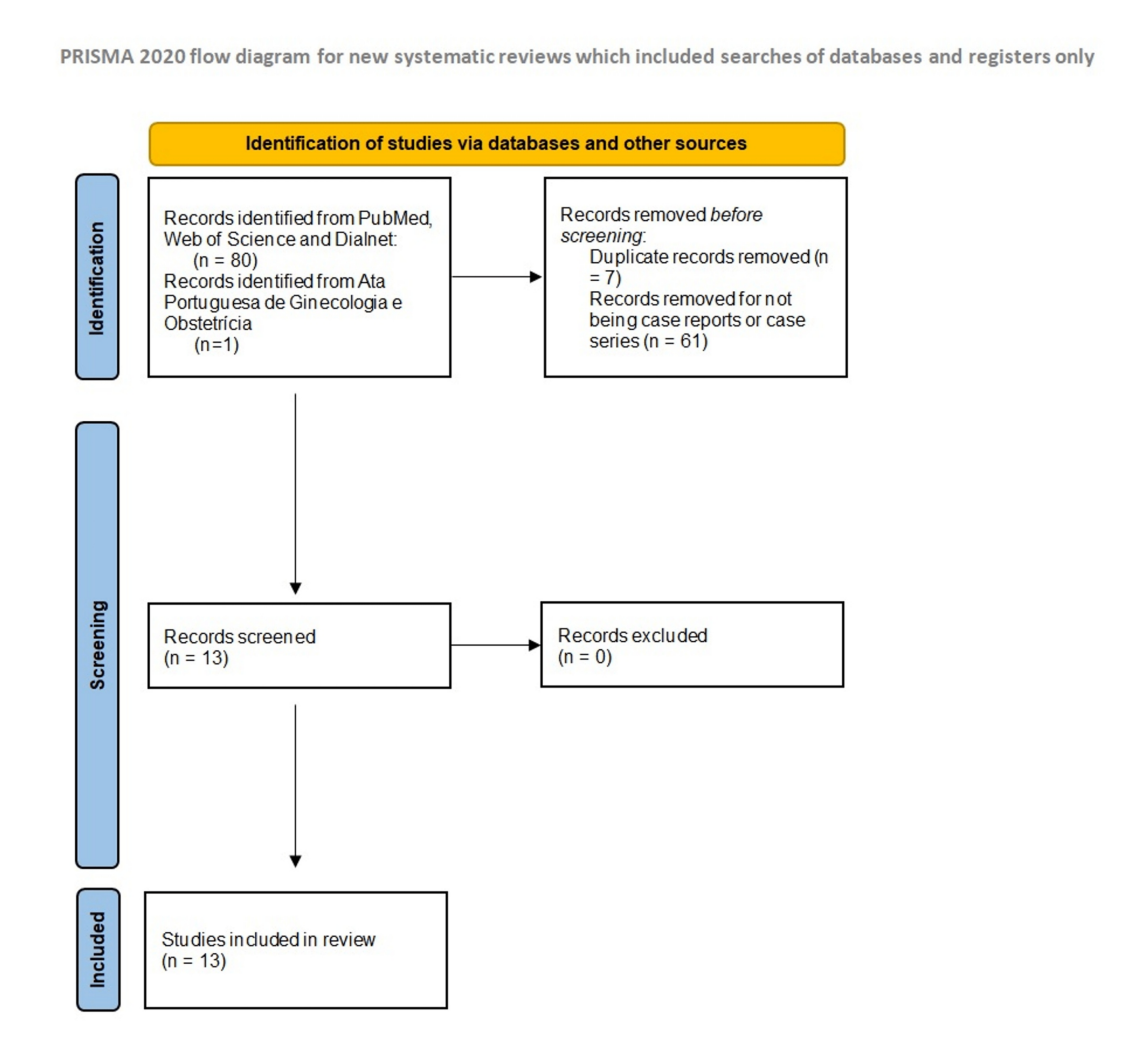
PRISMA flow diagram PRISMA, Preferred Reporting Items for Systematic Reviews and Meta-Analyses

Following the Preferred Reporting Items for Systematic Reviews and Meta-Analyses (PRISMA) (Figure 1), the authors conducted a literature search of PubMed, Web of Science, and Dialnet, as well as the journal Ata Portuguesa de Ginecologia e Obstetrícia (Journal of the Portuguese Societies of Gynecology and Obstetrics). The authors searched for case reports or case series in Portuguese, English, French, or Spanish using the combination of the keyword “pregnancy” and any of the following: “McArdle's Disease”, “Type V Glycogenosis”, or “Myophosphorylase”, available from January 1, 1970, until February 18, 2023. Thirteen articles corresponded to the search criteria: one of them was a case series with 14 patients and 20 pregnancies and the other 12 articles were case reports. The obstetric index, comorbidities, gestational age, description of pregnancy evolution, mode of delivery, intrapartum pharmacological measures, and clinical evolution in the postpartum period were analyzed in a retrospective descriptive study. No case series or case report articles were excluded, although not all of them covered all these topics. The statistical analysis was performed using Microsoft Excel (Microsoft Corp., Armonk, NY).

Results

A total of 26 patients with McArdle’s disease and 37 pregnancies (one of them a twin pregnancy) were analyzed in a retrospective descriptive analysis. Their demographic analysis is illustrated in Table 1.

**Table 1:** Demographic characterization of the studied population BMI, body mass index

Article	Article type	Number of patients	Number of pregnancies	Mother's age	Number of previous pregnancies	Medical co-morbidities
Stopp et al. [3]	Case report	1	1	Not mentioned in the article	0	Crohn's disease
Giles and Maher [4]	Case report	1	2	30	0	None
				Not mentioned in the article	1	None
Quinlivan et al. [5]	Case series	14	20	Not mentioned in the article	Not mentioned in the article	Not mentioned in the article
Canedo Carballeira et al. [6]	Case report	1	1	30	0	None
Coleman [7]	Case report	1	1	25	0	None
Samuels and Coleman [8]	Case report		1	31	1	None
Cochrane and Alderman [9]	Case report	1	1	21	0	None
Findlay et al. [10]	Two case reports	2	3	18	0	None
				24	1	None
				28	0	None
Lepoivre et al. [11]	Case report	1	2	27	0	Dilated cardiomyopathy
				29	1	Dilated cardiomyopathy
McMillan et al. [12]	Case report	1	1	23	0	Grade 2 obesity (BMI 39 kg/m^2^)
Nash et al. [13]	Case report	1	1	34	0	Arterial hypertension, gastroesophageal reflux, dyslipidemia, chronic pain, grade 3 obesity (BMI 48 kg/m^2^), irritable bowel syndrome
Vilela et al. [14]	Case report	1	1	35	0	Recurrent tonsillitis and allergic rhinitis
Melo et al. [15]	Case report	1	2	33	0	None
				35	1	None

Age

The mean age of the 16 patients whose age was mentioned was 27.75 years (minimum 18 years, maximum 35 years) [3,4,5-14].

Obstetric Index

In all the reports included, the obstetric index was mentioned; 13 pregnancies were first pregnancies [3,7,9-15] and five were second pregnancies [4,8,10,11,15]. Previous abortions were not described.

Comorbidities

In addition to McArdle's disease, some of the patients had other diseases: one patient had Crohn's disease [3]; one had dilated cardiomyopathy [11]; one had grade II obesity [12]; one had arterial hypertension, dyslipidemia, gastroesophageal reflux disease, and grade III obesity [13]; and one had recurrent tonsillitis and allergic rhinitis [14].

Clinical Evolution During Pregnancy

In most cases, there was no mention of complications (Table 2).

**Table 2:** Complications and improvement in symptoms during pregnancy CK, creatinine kinase

Article	Number of patients	Number of pregnancies	Pregnancy complications related to McArdle's disease	Pregnancy complications not related to McArdle's disease	Improvement of McArdle's symptoms during pregnancy
Stopp et al. [3]	1	1	Rhabdomyolysis at 25 weeks triggered by a gastroenteritis (maximum CK 7,800 U/L	None	Not mentioned in the article
Giles and Maher [4]	1	2	Rhabdomyolysis at 7 weeks after swimming (maximum CK 23,500 U/L)	None	Not mentioned in the article
			None	None	Not mentioned in the article
Quinlivan et al. [5]	14	20	One had mild myoglobinuria. Remainder had no antepartum complications.	None	Two patients referred worsening of their symptoms but the remainder referred an improvement
Canedo Carballeira et al. [6]	1	1	None	Idiopathic thrombocytopenia	Not mentioned in the article
Coleman [7]	1	1	None	Arterial hypertension; Preterm delivery	Not mentioned in the article
Samuels and Coleman [8]		1	None	Arterial hypertension	Not mentioned in the article
Cochrane and Alderman [9]	1	1	None	None	None
Findlay et al. [10]	2	3	None	Gestational diabetes	Not mentioned in the article
			None	Gestational diabetes	Not mentioned in the article
			None	None	Not mentioned in the article
Lepoivre et al. [11]	1	2	None	None	Not mentioned in the article
			None	Acute heart failure; symptomatic at 35 weeks’ gestation	Not mentioned in the article
McMillan et al. [12]	1	1	Rhabdomyolysis at 23 weeks, unknown trigger (maximum CK 33,000 U/L)	None	Yes
Nash et al. [13]	1	1	Rhabdomyolysis at 10 weeks triggered by an upper respiratory tract infection	Gestational diabetes, preeclampsia	Not mentioned in the article
Vilela et al. [14]	1	1	None	None	Increased tolerance to exercise; improvement of muscle cramps and no further episodes of myoglobinuria and rhabdomyolysis, with CK levels lower than preconception values
Melo et al. [15]	1	2	None	None	Improvement of symptoms and normalization of CK during pregnancy
			None	None	None

Some cases of rhabdomyolysis during pregnancy have been observed. Giles et al. [4] reported an episode of rhabdomyolysis at seven weeks of gestation, after swimming, with a maximum creatinine kinase (CK) level of 23,500 U/L. Nash et al. [13] reported an episode of rhabdomyolysis at 10 weeks of gestation, after an upper respiratory infection, with a maximum CK level of 35,763 U/L. McMillan et al. [12] reported the case of a patient at 23 weeks of gestation with no evident triggering factor, with a maximum CK level of 33,000 U/L. Stopp et al. [3] reported an episode of rhabdomyolysis at 25 weeks of gestation, triggered by acute gastroenteritis, with a maximum CK level of 7,800 U/L.

Other complications during pregnancy apparently not related to McArdle's disease were also described: two of the pregnant women developed gestational diabetes, and one of them developed it in both pregnancies [11,13]; one had pre-eclampsia (during pregnancy she also developed gestational diabetes and had rhabdomyolysis secondary to a respiratory infection) [13]; one had worsening complaints of heart failure (patient with known dilated cardiomyopathy) [11]; and one developed idiopathic thrombocytopenia, with a minimum value of 72,000/μL platelets, without hemorrhagic dyscrasia, which normalized after delivery [6].

In the data reported by Quinlivan et al. [5], two women reported worsening of myalgia during pregnancy, while the rest reported symptomatic improvement. There is also reference to the improvement of the usual symptoms in four other patients [6,12,14,15]. One of them mentioned improvement of tolerance to exertion [15].

Type of Delivery

Of the 37 pregnancies analyzed, 16 (43.2%) ended in cesarean section (Table 3). The indications mentioned for cesarean sections were lack of progression of labor (four), previous cesarean section (two), fetal-pelvic incompatibility (two), maternal pathology (two pregnancies in the patient with heart failure due to dilated cardiomyopathy, and one in a patient with arterial hypertension whose fetus was underweight), changes in fetal heart rate on the cardiotogram (two), and mother’s preference (one). Two cesareans were performed urgently, without specifying the reason. The remaining 22 deliveries were vaginal, of which five were instrumented (two requiring a suction cup, which is used to minimize maternal effort during the second stage, and three using forceps).

**Table 3:** Data regarding the delivery and neonatal outcomes

Article	Number of patients	Number of pregnancies	Proposed mode of delivery	Mode of delivery (indication)	Gestational age at delivery (weeks)	Anesthetic modality for delivery	Neonatal outcomes
Stopp et al. [3]	1	1	Cesarean section (mother's request)	Cesarean section (mother's request)	>38	Epidural	Live male, birth weight 2,870 g, Apgar score 9/9, cord arterial pH 7.31
Giles and Maher [4]	1	2	Vaginal	Cesarean section (lack of progress in the second stage)	>38	Epidural	Live male, birth weight 3,900 g, Apgar score 9/9, cord arterial pH 7.32
			Cesarean section	Cesarean section (previous cesarean section)	>38	Unknown	Unknown
Quinlivan et al. [5]	14	20	1 cesarean section, 20 vaginal	17 vaginal; 2 vaginal using forceps; 2 urgent cesarean sections; 1 cesarean section due to previous cesarean section	Unknown	Unknown	Unknown
Canedo Carballeira et al. [6]	1	1	Vaginal	Suction cup (suction cup to minimize maternal effort)	>38	Epidural	Live male, birth weight 2,740 g, Apgar score 7/9, cord arterial pH 7,12.
Coleman [7]	1	1	Cesarean section	Cesarean section (Hypertension and fetal low weight)	33	General anesthesia (mother's request)	Live male, birth weight 2,300 g, Apgar score 7/10
Samuels and Coleman [8]		1	Cesarean section	Cesarean section	Unknown	Epidural	Unknown
Cochrane and Alderman [9]	1	1	Vaginal	Vaginal (forceps)	>38	Epidural	Live female, birth weight 3,200 g, Apgar score 10
Findlay et al. [10]	2	3	Vaginal	Vaginal (suction cup to minimize maternal effort)	>38	Epidural	Unknown
			Vaginal	Cesarean section (arrested progression of labor, drop in fetal heart rate)	>38	Epidural	Unknown
			Vaginal	Cesarean section (arrested progression of labor at 9-cm dilation, fetal distress)	>38	Epidural	Unknown
Lepoivre et al. [11]	1	2	Cesarean section	Cesarean section (mother with dilated cardiomyopathy)	37	Epidural	Unknown
			Cesarean section	Cesarean section (mother with dilated cardiomyopathy)	35	General anesthesia	Live male, birth weight 2,540 g
McMillan et al. [12]	1	1	Vaginal	Vaginal	Unknown	Epidural	Live infant, Apgar score 8/9
Nash et al. [13]	1	1	Vaginal	Cesarean section (1st stage arrest)	37 + 5 days	Epidural	Unknown
Vilela et al. [14]	1	1	Vaginal	Cesarean section (non-reassuring fetal heart rate CTG)	>38	Epidural	Live male, birth weight 3,180 g, Apgar score 9/10
Melo et al. [15]	1	2	Cesarean section (fetopelvic disproportion)	Cesarean section	>38	Unknown	Unknown
			Cesarean section (fetopelvic disproportion)	Cesarean section	>38	Unknown	Unknown

Anesthesia

In the articles where it was mentioned, most patients received epidural anesthesia [3,4,6-14]. Only two case reports described patients who underwent general anesthesia: one due to cardiomyopathy [11] and the other at the patient's request [7].

Clinical Evolution in the Postpartum Period

There have been some reported cases of rhabdomyolysis after childbirth. In the data reported by Quinlivan et al. [5], there was only mention of one patient who reported myoglobinuria after the delivery of her third child (the postpartum day or CK values were not specified). Findlay et al. report one patient who developed rhabdomyolysis 9 hours after delivery, progressing to lower limb compartment syndrome, requiring fasciotomy (maximum CK 18,425 U/L, with subsequent normalization of CK). McMillan et al. [12] reported that one patient complained of shoulder pain and CK elevation to 28,500 U/L on the day of delivery. Five patients had an asymptomatic increase in CK (it is unknown whether this evaluation was carried out in all patients) [10,13-15].

Discussion

Type V collagen storage disease, also known as McArdle's disease, is a rare genetic condition inherited with an autosomal recessive pattern and caused by mutations in the gene that encodes the enzyme myophosphorylase [5]. This enzyme plays a role in glucose metabolism, namely in the transformation of glycogen into glucose. The deficiency of this enzyme results in the accumulation of glycogen in the muscle tissues and an inability to produce energy through glycogen storage [16,17].

Symptoms, such as asthenia, myalgias, cramps, and intolerance to exertion, usually begin in adolescence or adulthood. Intolerance to exertion that occurs shortly after starting physical activity and the “second wind” phenomenon (improvement of symptoms after about 10 minutes of recovery) are important clinical findings to take into account in the diagnosis of this disease. Cramps, when persistent, may be accompanied by rhabdomyolysis, with marked elevation of CK and myoglobinuria, which can lead to acute kidney injury [18]. The diagnosis can be established by genetic testing or by muscle biopsy that confirms the presence of collagen deposits [2].

Treatment is based on treating the symptoms and avoiding high-intensity physical activity. Some changes in the diet, such as the ingestion of carbohydrates before beginning a more intense physical activity, may delay the onset of symptoms [19].

Fertility is not affected in McArdle's disease, and there are some reported cases of pregnancies in women with this condition. Apparently, this disease does not represent a risk factor for complications related to pregnancy or childbirth. The cases of rhabdomyolysis described during pregnancy occurred in the context of infectious complications [3,13] and physical exercise [4], and only one occurred without apparent cause [12].

Symptomatic improvement, with a decrease in CK, is frequently described during pregnancy (16 of the 26 women described this in our review) [5,6,12,14,15]. This improvement can be explained by the improvement in muscle perfusion during pregnancy, with greater oxygen delivery and less need for anaerobic glycolysis, along with other metabolic alterations of pregnancy [9,20]. The insulin resistance of pregnancy also reduces lipolysis, leading to a high concentration of serum fatty acids, which function as an alternative energy source for skeletal muscle during pregnancy [21]. Insulin resistance increases significantly in the second and third trimesters of pregnancy, which may explain why the described cases of rhabdomyolysis occurred only in the first and second trimesters (7th, 10th, 23rd, and 25th weeks of gestation) and the reduction of CK during the third trimester [22]. Furthermore, during pregnancy, women are expected to avoid behaviors that trigger episodes of rhabdomyolysis, such as intense physical exercise or drinking alcohol.

McArdle's disease does not affect the uterine smooth muscle, and thus, uterine contractions are not affected by this condition and therefore should not, by itself, constitute an indication for cesarean section [6]. In this review, there were 16 cesarean sections and 23 vaginal deliveries (five of which were instrumented).

The use of adequate and early analgesia during labor is essential to avoid skeletal muscle effort and the emotional stress of childbirth, which can enhance rhabdomyolysis [23]. Based on the available literature, the administration of intravenous glucose helps in the prevention of myopathy by preventing the breakdown of glycogen reserves [4,7,9,12]. The second stage of labor in particular should be monitored and shortened as much as possible, as repetitive contraction of the abdominal wall muscles can lead to rhabdomyolysis, with one reported case of rhabdomyolysis after delivery in a patient with a second stage of prolonged labor (159 minutes) [12]. Of the 14 cases in which the anesthetic modality used during delivery was described, an epidural was chosen in 12 cases [3,4,6-14]. Patients with McArdle's disease have an increased risk of malignant hyperthermia after the administration of some anesthetics used in neuromuscular blockade [4]. Other anesthetic problems include rhabdomyolysis, myoglobinuria, or acute kidney injury. In cases where cesarean section is necessary, locoregional anesthesia is preferable [24]. During the postpartum period, there were three cases of symptomatic rhabdomyolysis [5,10,12], one of whom developed compartment syndrome [10]. In this case, the compartment syndrome was attributed to the use of an alternating compression system and compression stockings; moreover, dextrose was not administered because the patient had gestational diabetes. Five patients with an asymptomatic increase in CK have also been described (it is unknown whether this assessment was carried out in all patients) [10,13-15]. We did not find specific protocols in the literature for the surveillance of pregnant women with McArdle's disease. Currently, there is no scientific evidence to recommend prenatal genetic tests, as it is an autosomal recessive disease, usually without severe or debilitating symptoms [1]. Due to the scarce description in the literature of cases of pregnant women with McArdle's disease, it remains unclear whether fetuses are affected by episodes of rhabdomyolysis, although it has already been demonstrated that human placentas are not permeable to maternal myoglobin, which conveys some reassurance regarding the impact of crises on fetuses [25].

This is the largest revision published to date on the impact of McArdle's disease on pregnancy, delivery, and postpartum period. The authors acknowledge some study limitations, namely the fact that it was written based on previously published case reports, the authors only had access to what was published, and there is a lack of information on the reason for many of the cesarean sections performed and the anesthetic modality used for delivery and neonatal outcomes. Also, CK levels were not measured routinely in all patients during pregnancy and postpartum.

Although there are few reported cases of pregnant women with McArdle's disease, follow-up during pregnancy, delivery, and postpartum is essential to prevent complications. In the authors’ opinion, these patients should be followed up by multidisciplinary teams with expertise in high-risk pregnancies.

## Conclusions

Pregnancy does not seem to be associated with a greater number of episodes of rhabdomyolysis. Adequate hydration should be maintained, and physical exercise that is more intense than usual should be avoided. Intravenous glucose administration during delivery seems to reduce episodes of rhabdomyolysis, and positioning seems to be important so as not to compromise the circulation of any of the limbs (avoiding frequent blood pressure monitoring with the sleeve and the use of sequential limb compression systems). Epidural analgesia seems to be appropriate in most cases, and although vaginal delivery is usually the most indicated, the need to shorten the duration of labor should be considered due to the risk of rhabdomyolysis associated with prolonged contraction of the muscles of the abdominal wall.

There is no reference in any of the articles to the subsequent follow-up of the children, but in the case of an autosomal recessive disease, they have a 50% probability of being carriers of the disease, and sending them to a medical genetic consultation should be considered. Developing protocols for women with McArdle's disease, specifically designed for the period of pregnancy and the postpartum period, is essential for standardizing care.

## References

[1] Wright KE (2025). The McArdle Disease Handbook. https://www.glykogenose.de/download/McArdleDiseaseHandbook_Draft_v09_20_04_18.pdf.

[2] Santalla A, Nogales-Gadea G, Ørtenblad N, Brull A, de Luna N, Pinós T, Lucia A (2014). McArdle disease: a unique study model in sports medicine. Sports Med.

[3] Stopp T, Feichtinger M, Eppel W, Stulnig TM, Husslein P, Göbl C (2018). Pre- and peripartal management of a woman with McArdle disease: a case report. Gynecol Endocrinol.

[4] Giles W, Maher C (2011). Myophosphorylase deficiency (McArdle disease) in a patient with normal pregnancy and normal pregnancy outcome. Obstet Med.

[5] Quinlivan R, Buckley J, James M (2010). McArdle disease: a clinical review. J Neurol Neurosurg Psychiatry.

[6] Canedo Carballeira EM, Freire Vila E, Carballo Martíneza MJ, Iglesia López A (2008). Control del embarazo y manejo intraparto en la enfermedad de McArdle [Pregnancy control and management of labor in McArdle’s disease]. Progresos Obstet.

[7] Coleman P (1984). McArdle's disease. Problems of anaesthetic management for Caesarean section. Anaesthesia.

[8] Samuels TA, Coleman P (1988). McArdle's disease and caesarean section. Anaesthesia.

[9] Cochrane P, Alderman B (1973). Normal pregnancy and successful delivery in myophosphorylase deficiency (McArdle's disease). J Neurol Neurosurg Psychiatry.

[10] Findlay S, Liu D, Rijhsinghani A (2014). Acute compartment syndrome: clinical course and laboratory findings in pregnant patients with McArdle's disease. Pain Med.

[11] Lepoivre T, Legendre E, Pinaud M (2002). Anesthésie pour césarienne chez une patiente atteinte d’une maladie de McArdle et d’une cardiomyopathie dilatée familiale [Anaesthesia for caesarean section in a patient with MacArdle disease and hereditary dilated cardiomyopathy]. Ann Fr Anesth Reanim.

[12] McMillan BM, Hirshberg JS, Cosgrove SC (2019). McArdle disease causing rhabdomyolysis following vaginal delivery. Anaesth Rep.

[13] Nash CM, Shetty N, Miller A, McCoy K (2022). McArdle disease and pregnancy: a case report and scoping review of pregnancy outcomes. Obstet Med.

[14] Vilela F, Martins R, Edral A (2019). Doença de McArdle e Gravidez. Acta Obstet Ginecol Port.

[15] Melo J, Gomes B, Leita J, Agúndez M (2015). McArdle disease and pregnancy. Galicia Clin.

[16] Llavero F, Arrazola Sastre A, Luque Montoro M, Gálvez P, Lacerda HM, Parada LA, Zugaza JL (2019). McArdle disease: new insights into its underlying molecular mechanisms. Int J Mol Sci.

[17] Nogales-Gadea G, Godfrey R, Santalla A (2016). Genes and exercise intolerance: insights from McArdle disease. Physiol Genomics.

[18] Martin M, Lucia A, Arenas J (2006). Glycogen storage disease type V. GeneReviews® [Internet].

[19] Andersen ST, Vissing J (2008). Carbohydrate- and protein-rich diets in McArdle disease: effects on exercise capacity. J Neurol Neurosurg Psychiatry.

[20] Lucia A, Nogales-Gadea G, Pérez M, Martín MA, Andreu AL, Arenas J (2008). McArdle disease: what do neurologists need to know?. Nat Clin Pract Neurol.

[21] Sivan E, Homko CJ, Chen X, Reece EA, Boden G (1999). Effect of insulin on fat metabolism during and after normal pregnancy. Diabetes.

[22] Catalano PM, Huston L, Amini SB, Kalhan SC (1999). Longitudinal changes in glucose metabolism during pregnancy in obese women with normal glucose tolerance and gestational diabetes mellitus. Am J Obstet Gynecol.

[23] Brady S, Godfrey R, Scalco RS, Quinlivan RM (2014). Emotionally-intense situations can result in rhabdomyolysis in McArdle disease. BMJ Case Rep.

[24] Yokoi A, Iwakura H, Fujimoto K (2015). [Anesthesia in a patient with McArdle disease] [Article in Japanese]. Masui.

[25] Rizzardi R, Castelli S, Porta C, Vitali F, Minerva M, Della Marta ME, Raimondi M (1997). [Postpartum myoglobin blood monitoring in newborns. Correlations with renal function in the first 48 hours of life] [Article in Italian]. Minerva Anestesiol.

